# Predictors of Late Mortality in Patients With Surgically Resected Cardiac Myxomas: A Single-Center Experience

**DOI:** 10.7759/cureus.20866

**Published:** 2022-01-01

**Authors:** Raluca Tulin, Roxana Carmen Geana, Mircea Robu, Vlad Anton Iliescu, Ovidiu Stiru, Reza Nayyerani, Andreea Simina Chibulcutean, Nicolae Bacalbasa, Irina Balescu, Adrian Tulin, Luminita Tomescu

**Affiliations:** 1 Anatomy and Embryology, Carol Davila University of Medicine and Pharmacy, Bucharest, ROU; 2 Endocrinology, Prof. Dr. Agrippa Ionescu Clinical Emergency Hospital, Bucharest, ROU; 3 Cardiac Surgery, C.C. Iliescu Emergency Institute for Cardiovascular Diseases, Bucharest, ROU; 4 Department of Cardio-Thoracic Pathology, Carol Davila University of Medicine and Pharmacy, Bucharest, ROU; 5 Visceral Surgery, Center of Excellence in Translational Medicine “Fundeni” Clinical Institute, Bucharest, ROU; 6 Obstetrics and Gynaecology, Carol Davila University of Medicine and Pharmacy, Bucharest, ROU; 7 Clinic of General Surgery, Prof. Dr. Agrippa Ionescu Clinical Emergency Hospital, Bucharest, ROU; 8 Anatomy, Carol Davila University of Medicine and Pharmacy, Bucharest, ROU; 9 Radiology, Prof. Dr. Agrippa Ionescu Clinical Emergency Hospital, Bucharest, ROU

**Keywords:** predictor, mortality, chronic kidney disease, long-term prognosis, cardiac myxomas

## Abstract

Background and objective

Myxomas are the most common cardiac tumors. This study aimed to analyze the possible risk factors associated with late mortality in this group of patients and assess long-term survival.

Methods

A retrospective study was conducted among patients who underwent myxomas resection between January 2008 and July 2019 in our service. The patients' preoperative, intraoperative, and postoperative data were analyzed. Multivariate logistic regression was performed to identify predictors of mortality at five years. The Kaplan-Meier curve and Cox proportion-adjusted survival curves were used to assess mortality at five and 10 years.

Results

A total of 108 patients with cardiac myxomas were identified. All cardiac tumors resected were confirmed as myxomas on histopathological examination. Ninety-six patients presented with left-side myxomas (94 left-atria and two left-ventricle) and 12 with right-side myxomas (11 right-atria, one right-ventricle); 78 of the tumors were capsulated, and 30 were sessile-papillary. The mean dimensions were 37 ±6.1 mm on the left side and 41 ±6.7 mm on the right side. Surgical excision was successful in all cases, with 25% requiring interatrial septum patch repair. Recurrence occurred in 2.77% of the patients. Multivariate logistic regression showed chronic kidney disease (CKD) (OR: 7.96, 95% CI: 1.469-43.125, p=0,016) to be an independent predictor for five-year mortality. The mean follow-up period was 7.13 ±2.965 years, and the Kaplan-Meier curve cumulative proportion survival of patients at five years and 10 years were 100% and 88.8%, respectively. There was no statistically significant difference in late-term survival between patients with and without CKD in the Cox proportion-adjusted survival curve (p=0.275).

Conclusions

Patients with myxomas have a good long-term prognosis following surgical resection. The multivariate logistic regression showed CKD to be an independent predictor of five-year mortality.

## Introduction

Cardiac myxomas are benign entities and account for half of all primary cardiac tumors [1]; they are often associated with completely asymptomatic presentations. The volume of these tumors can become life-threatening due to the obstruction of the mitral valve and consequent embolic events [2]. The myxomatous disease occurs in all age groups and both sexes. It is a sporadic or familial disorder, more frequently seen in women between the ages of 50 and 70 years, and has an approximate incidence of 0.5-1.0 cases per million inhabitants per year [3,4]. The clinical manifestations usually depend on the size of the myxoma and the anatomic position in the heart. Most symptoms are related to tumor-related obstructions of intracardiac blood flow, tumor-related embolic events, and systemic symptoms [5]. Tricuspid valve pathology, inferior extremities edema, and right heart failure have been reported to be the main complaints in patients with right atrial myxomas. Systemic embolic manifestations include stroke and myocardial infarction, and peripheral or visceral infarctions were observed to be the main symptoms in patients with left atrial myxomas. Neurological deficit or moderate sensory impairment, as an onset manifestation, may sometimes raise suspicion for a left cardiac myxoma. Both transthoracic echocardiography (TTE) and transesophageal echocardiography (TEE) are the mainstays of investigations, although the latter provides more precise information needed for the scheduling of surgery. TEE has a higher sensitivity than TTE, and a 100% specificity, revealing even small tumors that are only 1-3-mm in diameter. Cardiac myxomas are diagnosed sporadically in 90% of patients [6,7]. From the pathological point of view, two types of cardiac myxomas have been described: solid and papillary [7]. Papillary myxomas are characterized by soft formations that are friable during the excision. The recurrence rate in these tumors is higher compared with those presenting as solid types [8]. Surgical resection is the first-line therapy and is associated with good early results.

Previous studies on the topic have focused on clinical presentations, diagnosis, and surgical techniques. However, there is scarce data regarding long-term outcomes in patients presenting with cardiac myxomas that underwent surgical resection, especially considering that a significant number of these patients have important comorbidities.

## Materials and methods

We retrospectively reviewed our single-center experience among patients who underwent surgical treatment of cardiac myxomas. Between January 2008 and July 2018, 108 consecutive patients underwent surgery and resection of myxomas at this center. Preoperatively, all patients underwent TTE, TEE, and coronary angiography, which were performed in male patients over the age of 40 years and female patients aged over 45 years. Researchers confirmed that they had obtained ethical approval to conduct the study, as well as permission from the dataset owner to use the information in databases/repositories for the research they were conducting. The agreement was signed on 01.03.2021 and is registered with the number 1369.

The study was a retrospective analysis, and the hospital Ethics Committee approved it. All preoperative information, such as age, sex, weight, tumor location, comorbidities (including embolic symptoms), cardiac output [heart failure was classified as per the New York Heart Association (NYHA) scale], was retrieved for analysis. Also, postoperative and intraoperative variables such as type of surgical interventions (including tumor approach based on myxoma location) and additional cardiac surgical procedures were also noted. The European System for Cardiac Operative Risk Evaluation (EuroSCORE), histopathological information, and performance status were taken into account. The variables analyzed were retrieved from the patient's hospital charts. All patients had follow-up evaluations at regular intervals (at one month, six months, and annually after that) at our institution and underwent physical examination, electrocardiography, and TTE. Confirmation of the cardiac tumor was obtained by histopathological examination in all cases. Continuous variables are shown as mean ±standard deviation (SD). Categorical variables are shown as counts (n) and percentages (%). Multivariate logistic regression was performed to identify predictors of mortality at five years. Univariate and multivariate logistic regression were used to analyze predictors for late mortality. The Kaplan-Meier curve and Cox proportion-adjusted survival curves illustrated mortality at five and 10 years.

For the 12-year survival rate, data regarding death was obtained from the national registry of persons based on the patient’s unique national identification code.

## Results

A total of 108 patients with surgical resection of a cardiac myxoma were identified. Table 1 presents the baseline clinical characteristics of the study population.

**Table 1:** Baseline clinical characteristics of the study population HF: heart failure; NYHA: New York Heart Association functional classification; AF: atrial fibrillation; IHD: ischemic heart disease; MR: mitral regurgitation; TR: tricuspid regurgitation; PHT: pulmonary hypertension; COPD: chronic obstructive pulmonary disease; CKD: chronic kidney disease; OA: other arrhythmias; SD: standard deviation

Variables	Left side (n=96)	Right side (n=12)	Total (n=108)
Age in years (mean ±SD)	57.69 ±0.25	61.08 ±8.63	58.07 ±2.38
Sex (n/%) male, female	29/30.2, 67/69.8	8/66.7, 4/33.3	33/30.6, 75/69.4
Risk factors (n/%)			
Hypertension	59/60.2	7/50	66/61.1
Diabetes	8/8.2	2/14.3	10/9.3
Hyperlipidemia	59/60.2	9/64.3	68/63
BMI >25 kg/m^2^	14/14.3	2/14.3	16/14.8
Smoker	22/22.4	2/14.3	23/21.3
Comorbidities (n/%)			
HF: NYHA I, NYHA II, NYHA III, NYHA IV	9/9.4, 50/52.1, 31/32.3, 6/6.2	1/8.3, 2/16.7, 9/75, 0/0	10/9.3, 52/48.1, 40/37, 6/5.6
AF	17/17.3	5/35.7	22/20.4
IHD	7/7.1	2/14.3	9/7.4
MR	8/8.2	0/0	8/8.2
TR	10/10.2	0/0	10/10.2
PHT	18/18.4	3/21.4	21/19.4
OA	3/3.1	2/14.3	5/3.7
COPD	5/5.1	2/14.3	7/5.6
CKD	6/6.1	2/14.3	8/7.4

The mean age of the population was 58.07 ±2.38 years, and 69.4% belonged to the female gender. The initial study design included a follow-up protocol at five and 10 years after surgery but a significant number of patients were lost to follow-up (37.5% of the patients with 10 years of follow-up) due to lack of adherence.

Most of the patients had multiple cardiovascular risk factors, the most common being dyslipidemia in 63% of patients and essential arterial hypertension in 61.11% of patients, followed by smoking in 21.3% of patients. All patients had a form of heart failure according to the NYHA classification, the most frequent being NYHA II (48.1%). Left-side myxomas had an incidence of NYHA II of 52.1%, while in the right-side group, NYHA III was more frequent (75%). The incidence of perioperative atrial fibrillation (AF) was 20.4%, and 7.4% of the patients had ischemic heart disease. Mitral regurgitation and tricuspid regurgitation were present in 7.4% and 9.2%, respectively, and only in those with left-side localization.

Other arrhythmias accounted for five cases of which three were due to myxomas on the left side and two on the right side and were classified as follows: two cases with supraventricular extrasystoles (SVES), two cases with ventricular extrasystoles (VES), and one case with atrioventricular block first degree.

Chronic obstructive pulmonary disease was present in 5.6%, and 19.4% had pulmonary hypertension. Chronic kidney disease (CKD) was found in 7.4% of cases. The initial clinical presentation varied and is listed in Table 2.

In the study population, eight patients (four males and four females) were confirmed to have coronary disease. Of these, three cases required percutaneous coronary intervention (PCI) with drug-eluting stent (DES), and in the other five cases, coronary artery bypass graft (CABG) was performed concomitantly with the excision of the myxoma.

Case 1: 60% stenosis on the left anterior descending artery (LAD) I. Treatment: PCI with 1 x DES on LAD.

Case 2: 70% stenosis on LAD I and 60% on the right coronary artery (RCA). Treatment: PCI with 2 x DES on LAD and RCA.

Case 3: 60% stenosis on LAD I, 60% on the left circumflex coronary artery (LCX), and 30% stenosis on RCA. Treatment: PCI with 2 x DES on LAD and LCX.

Case 4: 85% stenosis on LAD II, 75% on LCX, and 80% stenosis on RCA. CABG with the left internal mammary artery (LIMA) on LAD, saphenous vein graft (SVG) on the obtuse marginal artery (OM) I, and SVG on RCA.

Case 5: 85% stenosis on LAD I. Treatment: CABG with LIMA on LAD.

Case 6: 90% stenosis on LAD II and 80% on RCA. Treatment: CABG with LIMA on LAD and SVG on RCA.

Case 7: 80% stenosis on RCA. Treatment: CABG with SVG on RCA.

Case 8: 90% stenosis on LM. Treatment: CABG with LIMA on LAD, SVG on OM I, and SVG on the intermediate artery.

**Table 2:** Clinical presentation in the study population

Clinical presentation	Left location (n=96)	Right location (n=12)	Total (n=108)
Asymptomatic (n/%)	39/40.62	6/50	45/41.66
Dyspnea (n/%)	33/34.37	5/41.66	38/35.18
Fatigability (n/%)	24/25	2/16.66	26/24.07
Palpitation (n/%)	16/16.66	2/16.66	18/16.66
Chest pain (n/%)	11/11.45	0/0	11/10.18
Syncope (n/%)	7/7.29	1/8.33	8/7.40
Vertigo (n/%)	4/4.16	0/0	4/3.70
Systemic embolic symptoms (n/%)	28/29.2	1/8.33	29/26.9

In our study, 45 (41.66%) patients were asymptomatic. More than a third of the patients had dyspnea at presentation. Fatigability (24.07%), palpitations (16.66%), and chest pain (16.66%) were also common. Systemic embolic symptoms occurred in 14 (14.58%) patients before myxoma resection.

Surgical technique

All 108 patients underwent surgery and resection of the cardiac myxomas, which were conducted via median sternotomy. Cardiopulmonary bypass with aortic and bicaval cannulation was used in all cases. Cardiac arrest was provided using antegrade cold blood cardioplegia. Extra care had been taken to minimize the heart's manipulation when passing inferior caval snare and in inferior vena cava cannulation to avoid tumor embolization. The surgical approach was performed according to the localization of the myxoma. Left atrial myxomas were approached by left atriotomy in the interatrial groove atrium. Right atrial myxomas were resected through a right atriotomy. The incision to excise the left ventricular myxomas was made at the apical left ventricle parallel to the left anterior descending coronary artery in order to avoid vessel damage. After the radical excision of the myxomas, including the implantation base for all cases, the cardiac chambers involved were irrigated with saline solution to prevent possible micro embolisms. Additional cardiac surgical procedures required were as follows: coronary bypass surgery in five patients (4.6%), mitral valve replacement in 10 patients (9.3%), atrium septal repair in 27 patients (25%), and tricuspid valve annuloplasty in 11 patients (10.2%). The main surgical characteristics are listed in Table 3.

**Table 3:** Surgical characteristics of the study population LA: left atrium; LV: left ventricle; RA: right atrium; RV: right ventricle; CABG: coronary artery bypass grafting; MVR/MVP: mitral valve replacement/mitral valve plasty; TV: tricuspid valve; SD: standard deviation

Variables	Left side (n=96)	Right side (n=12)	Total (n=108)
EuroSCORE (mean ±SD)	5.20 ±0.48	6.08 ±1.88	5.30 ±0.46
Resection (n/%)			
LA	94/87		
LV	2/1.9		
RA		11/10.2	
RV		1/0.9	
Associated procedures (n/%)	36/37.9	5/41.7	43/39.8
CABG	5/5.3	0/0	5/4.6
MVR/MVP	10/10.5	0/0	10/9.3
Atrial septum repair	23/24.2	4/33.34	27/25
TV annuloplasty	8/8.4	3/25	11/10.2
Bypass time (mean ±SD)	55.8 ±6.44	56.91 ±24.99	55.78 ±6.15
Aortic clamp time (mean ±SD)	36.65 ±5.16	38.25 ±25.12	36.75 ±5.14

Postoperative data 

The mean EuroSCORE was 5.2 ±0.48 in patients with left-sided myxomas and 6.08 ±1.88 in patients with right-sided myxomas. Postoperative cerebrovascular embolic events were observed only in left-sided myxomas [in three (2.88%) patients]. The mean in-hospital stay was of 8.25 ±2.73 days. Echocardiographic follow-up was available in 98.1% of patients. Three (2.77%) patients experienced recurrences, all in left-sided myxomas, detected at 24, 36, and 40 months after the myxoma resection. One patient died from cardiogenic shock on the third postoperative day. The main postoperative characteristics are listed in Table 4.

**Table 4:** Postoperative characteristics of the study population AVB: atrioventricular block; AF: atrial fibrillation; ICU: intensive care unit; SD: standard deviation

Variables	Left side (n=96)	Right side (n=12)	Total (n=108)
Days in ICU (mean ±SD)	2.79 ±1.46	4.12 ±3.22	2.89 ±0.31
Early complications (n/%)	23/24.2	3/25	26/24.3
New onset of AF	11/11.5	1/8.3	12/11.1
AVB	12/12.5	2/16.7	14/13
Cerebral embolization	3/3.12	0/0	3/2.77
Wound dehiscences (n/%)	3/3.12	0/0	3/2.77
Hospital stay (days, mean ±SD)	8.10 ±2.55	9.25 ±4.13	8.25 ±2.73
Myxomas recurrence (n/%)	3/3.12	0/0	3/2.77
Mortality within 30 days of the surgery (n/%)	1/1.05	0/0	1/0.92
Recurrence (n/%)	3/3.12	0/0	3/2.77

Histopathologic examination was consistent with the diagnosis of myxoma in all 108 patients. The macroscopic appearance was of a sessile tumor in 30 patients and a solid tumor in 78 patients (Table 5).

**Table 5:** Histopathological aspects in patients with cardiac myxoma SD: standard deviation

Appearance	Left heart location (n=96)	Right heart location (n=12)
Solid-capsulated (n/%)	66/68.75	12/100
Sessile-papillary fragmentated	30/31.25	0/0
Dimension (mm, mean ±SD)	37.0 ±6.1	41.0 ±6.7

The mean length (TTE examination) of the tumor was 37.0 ±6.1 mm in left myxomas and 41.0 ±6.7 mm in right myxomas. The sessile histopathological form of left-sided myxomas was encountered in all cases of embolic onset of symptomatology, in myxoma recurrence, and in postoperative cerebrovascular complications.

Predictors of late mortality 

Univariate and multivariate logistic regression analyses were performed for predictors of late mortality (Table 6).

**Table 6:** Univariate and multivariate logistic regression for predictors of late mortality CKD: chronic kidney disease; OR: odds ratio; CI: confidence interval

	Univariate analysis		Multivariate analysis	
	OR (95% CI)	P-value	OR (95% CI)	P-value
CKD	6.067 (1.24-29.65)	0,02	7.96 (1.46-43.12)	0.01
Aortic cross-clamp	1.01 (1-1.03)	0.04	0.99 (0.92-1.05)	0.77
Bypass time	1.01 (1-1.03)	0.024	1.02 (0.97-1.08)	0.32

Univariate logistic regression identified CKD, aortic cross-clamp time, and bypass time as such. After multivariate analysis, only the presence of CKD was identified as an independent predictor of late mortality (OR: 7.96, 95% CI: 1.46-43.12, p=0.01).

Besides CKD, aortic cross-clamp, and bypass time, postoperative atrial fibrillation (POAF) and postoperative neurologic complications due to cerebral embolization were analyzed as predictors of late mortality in our study population, but these were not found to have an impact on survival among the population.

Survival analysis

**Figure 1:**
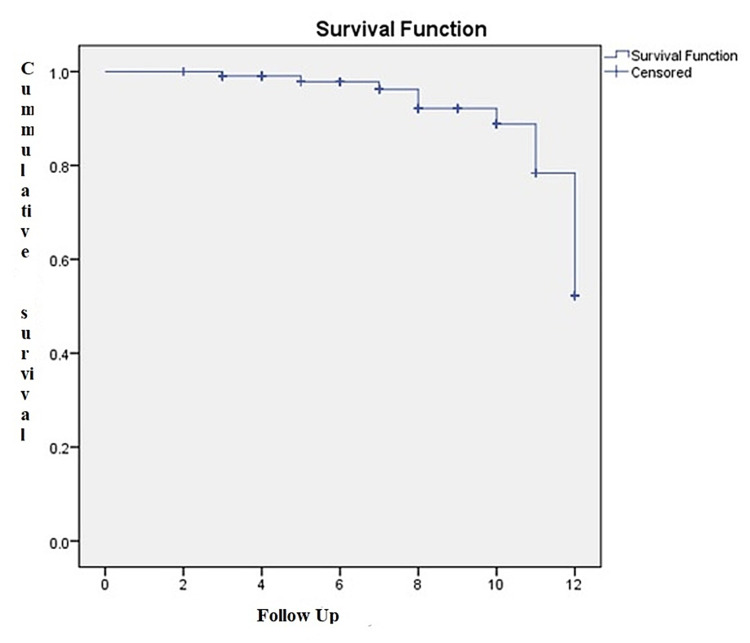
The overall survival among the total 108 patients

The mean follow-up period was 7.13 ±2.96 years; patients with CKD had a mean follow-up of 8.14 ±3.80 years, and it was 7.06 ±2.90 for the ones without CKD. The overall survival at five, 10, and 12 years was 97.8%, 88.8%, and 52.3%, respectively (Figure 1).

Our initial study design included a follow-up protocol at five and 10 years postoperative, and after this period, we lost a significant number of patients to follow-up (37.5% of the patients with 10 years of follow-up) due to lack of adherence to the follow-up protocol, which influenced the results. For the 12-year survival rate, data regarding death were obtained from the national registry of persons based on the patient’s unique national identification code.

**Figure 2:**
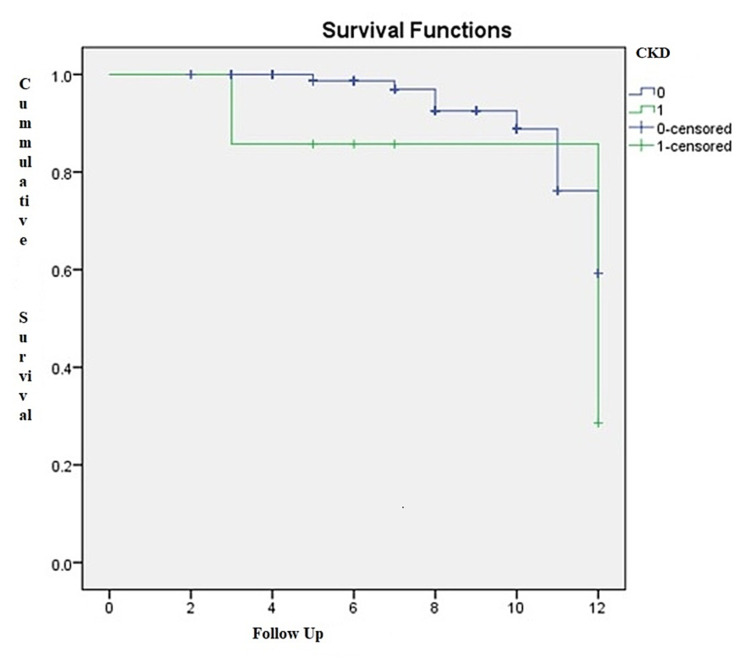
Cox proportion-adjusted survival curve (log-rank p=0.27) CKD: chronic kidney disease

Survival at five, 10, and 12 years for patients without CKD was 98.7%, 88.8%, and 59.2%, respectively. For patients with CKD, survival at 12 years was 28.6% (Figure 2).

**Figure 3:**
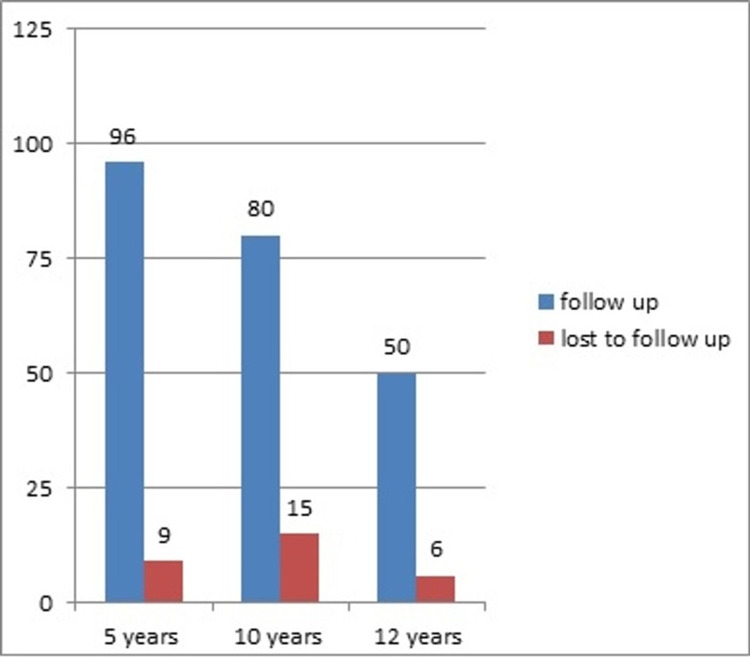
Flow chart regarding the number of patients lost to follow-up

There was no statistically significant difference in late-term survival between patients with and without CKD in the Cox proportion-adjusted survival curve (p=0.27) (Figure 2). During the 12-year period, 30 patients were lost to follow-up: nine patients after five years, 15 after 10 years, and six patients after 12 years (Figure 3).

The causes of in-hospital mortality were mentioned, and for the rest of the patients whose death occurred outside the clinic, the national registry of persons was consulted. The following data were obtained: 17.3% of patients died of myocardial infarction, 19.23% of stroke and comma unrelated to the myxoma resection surgery, 9.61% of arrhythmias, 7.69% of end-stage heart failure, and 46.15% of the patients died outside of the hospital environment due to natural causes.

## Discussion

Cardiac myxoma is a rare primary benign tumor with a variable clinical presentation. TTE provides a precise method for diagnosis and is a very helpful tool for follow-up [9].

The mean age in our cohort at presentation was 57.69 years for males and 61.08 years for females, as shown in Table 1. In our study, we also included patients over 70 years old, the maximum age being 86 years. In the initial study follow-up plan, our primary endpoints were late mortality outcomes at five and 10 years postoperatively, taking into consideration the mean survival age in the male general population, which is 72 years in our country. The accelerated decrease in survival rate at 12 years was due to the withdrawal of a significant number of patients from follow-ups and due to demographic evolution.

In specific cases, complete resection associated with mitral valve replacement or CABG has curative effects. Surgical resection provides the best results both in the short term and long term as has been demonstrated by Bakaeen et al. in their study on 85 patients with primary cardiac tumors [10]. Echocardiographic examination in the long-term follow-up is recommended for all patients as it is cheap and readily available although it has one drawback: it is operator-dependent [11]. The prevalence of CKD has been rising at the level of an epidemic and is mainly associated with an increase in diabetes mellitus and hypertension, two of the main causes of renal impairment [12,13]. CKD is associated with an increased risk of cardiovascular complications [14-16]. This may be attributed to inadequate blood pressure control, accelerated atherosclerosis, and a pro-inflammatory status as some of our results demonstrated. Further cohort studies are required to confirm these aspects. Besides CKD, aortic cross-clamp, and bypass time, we also analyzed POAF and postoperative neurologic complications due to cerebral embolization but they were not found to be predictors of late mortality in our study population.

CKD is a known risk factor for ischemic heart disease with a significant risk of mortality and morbidity [14-16] in these patients; however, little is known regarding the survival impact on patients with heart tumors. In our study, CKD was an independent predictor of five-year mortality in patients with surgical resection of a cardiac myxoma, but there was no statistically significant difference in late-term survival between patients with and without CKD. Although the attention is often focused on the cardiovascular system in patients with myxoma as in our study, other components also need to be evaluated in the long term.

All healthcare professionals should monitor and evaluate long-term renal function using the glomerular filtration rate in patients with heart disease. The correct assessment of the relationship between the two diseases and intervention is necessary to improve the prognosis of these patients. There are scarce data regarding the factors that influence late survival in patients with surgical resection of myxomas. One study focused on POAF as a predictor of late mortality in patients with surgical resection of myxoma but failed to demonstrate a significant difference in late survival between patients with and without this complication [17].

In our study, POAF was not a predictor of late mortality. Although rare, perioperative embolism could affect the outcomes in patients; however, this finding is based on singular cases and cannot be used as an independent factor [18]. As demonstrated in our study, only three patients had postoperative neurological complications due to embolization. The sessile pathological subtype was found in our series in all patients with preoperative systemic embolic symptoms as well as in patients who developed postoperative cerebrovascular complications. The broader base can influence the rate of emboli formation along with platelet volume, platelet count, and tumor location [19].

Also, size seems to play a role in the frequency of emboli formation, especially for tumors above <4.5 cm in diameter as demonstrated by Wang et al. [18]. Postoperative neurologic complications due to cerebral embolization were not a predictor of late mortality in our study population [20-21]. Further studies are necessary to evaluate the risk factors of late mortality in patients with heart tumors.

The primary limitation of the study is that it was retrospective in nature. The researchers could not control the exposure or outcome evaluation, and they needed to rely on accurate recordkeeping.

## Conclusions

Patients with myxomas have a good long-term prognosis following surgical resection. The sessile pathological subtype of left-sided myxomas was encountered in all cases with embolic symptoms, as well as recurrence. The postoperative cerebrovascular complications rate was also high in these patients. The sessile pathology subtype of myxomas should be considered an independent risk factor due to the likelihood of emboli formation. In our study, POAF was not a predictor of late mortality. Multivariate logistic regression showed CKD to be an independent predictor of five-year mortality. Keeping this aspect in mind, the renal function of all patients with cardiac pathology should be regularly monitored.
